# The identification of small molecule inhibitors of the plant inositol phosphorylceramide synthase which demonstrate herbicidal activity

**DOI:** 10.1038/s41598-019-44544-1

**Published:** 2019-05-30

**Authors:** Elizabeth C. Pinneh, John G. Mina, Michael J. R. Stark, Stephen D. Lindell, Peter Luemmen, Marc R. Knight, Patrick G. Steel, Paul W. Denny

**Affiliations:** 1 Department of Biosciences, Durham University, Durham, DH1 3LE UK; 2 Department of Chemistry, Durham University, Durham, DH1 3LE UK; 3 Centre for Gene Regulation and Expression, School of Life Sciences, University of Dundee, Dundee, DD1 5EH UK; 4 Bayer AG, Crop Science Division, Industriepark Höchst, 65926 Frankfurt am Main, Germany

**Keywords:** Plant sciences, Biochemistry

## Abstract

Resistance to 157 different herbicides and 88% of known sites of action has been observed, with many weeds resistant to two or more modes. Coupled with tighter environmental regulation, this demonstrates the need to identify new modes of action and novel herbicides. The plant sphingolipid biosynthetic enzyme, inositol phosphorylceramide synthase (IPCS), has been identified as a novel, putative herbicide target. The non-mammalian nature of this enzyme offers the potential of discovering plant specific inhibitory compounds with minimal impact on animals and humans, perhaps leading to the development of new non-toxic herbicides. The best characterised and most highly expressed isoform of the enzyme in the model-dicot *Arabidopsis, At*IPCS2, was formatted into a yeast-based assay which was then utilized to screen a proprietary library of over 11,000 compounds provided by Bayer AG. Hits from this screen were validated in a secondary *in vitro* enzyme assay. These studies led to the identification of a potent inhibitor that showed selectivity for *At*IPCS2 over the yeast orthologue, and activity against *Arabidopsis* seedlings. This work highlighted the use of a yeast-based screening assay to discover herbicidal compounds and the status of the plant IPCS as a novel herbicidal target.

## Introduction

First discovered in *Saccharomyces cerevisiae*, inositol phosphorylceramide synthase (IPCS or Aur1p in yeast) catalyses the transfer of phosphorylinositol from the phosphoglycerolipid phosphatidylinositol to the C-1 hydroxyl group of (phyto)ceramide, thereby generating the complex sphingolipid inositol phosphorylceramide (IPC)^1^. IPC is subsequently a precursor for the generation of the other, more complex, sphingolipids: mannosylinositol phosphorylceramide (MIPC) and mannosyldiinositol phosphorylceramide [M(IP)_2_C]^2^. These have been shown to be vital for the localization and endocytosis of plasma membrane proteins in *S. pombe*^3^. In addition, aside from maintaining the structural integrity of the plasma membrane, sphingolipids have been demonstrated to play crucial roles in a number of eukaryotic cell processes including apoptosis^4,5^, cell differentiation^6^, cell cycle arrest^7^, cell signalling^8^, angiogenesis^9^ and senescence^10^.

The sphingolipid biosynthetic pathway, and the enzymes involved, show conservation in all kingdoms of the Eukaryota up to the formation of dihydrosphingosine^11^. Subsequently there is divergence, dihydrosphingosine is *N*-acylated to produce dihydroceramide which is then desaturated to give ceramide in mammals and protozoa. In contrast, in plants and fungi, phytosphingosine, generated from the hydroxylation of dihydrosphingosine, is *N*-acylated to give phytoceramide. Subsequently, these intermediary metabolites are transported into the Golgi apparatus where sphingomyelin synthase catalyzes the production of sphingomyelin, the major sphingolipid in mammals, and IPCS generates IPC in plants, fungi and protozoa^11^.

This divergence in sphingolipid biosynthesis has been exploited to investigate the protozoal IPCS as a therapeutic target for the Neglected Tropical Diseases, Chagas disease^12–14^ and leishmaniais^15–18^. In plants, the activity of IPCS was first characterized in *Phaseolus vulgaris*^19^ and its role as a negative regulator of programmed cell death in plants was validated in *Arabidopsis thaliana*^20^ and *Eucalyptus grandis*^21^. In *Oryza sativa*, IPCS has been shown to play a role in plant response to abiotic stress, particularly in response to drought, cold and salinity^22^.

Despite the fact that hundreds of herbicides are widely used, these only exhibit 25 modes of action. In fact, merely 6 modes of action, targeting 5-enolpyruvylshikimate-3-phosphate (EPSP) synthase, acetolactate synthase (ALS), photosystem (PS) II, synthetic auxins, acetyl CoA carboxylase (ACCase) and cell division, acount for 75% of the herbicide market^23^. It has been over 30 years since a herbicide with a new mode of action was introduced onto the market and, with the growing problem of herbicide resistance^24^ and the destabilizing effect of climate change on crop yield^25^, it is now necessary to identify new herbicidal modalities to ameliorate the challenge of feeding a rapidly increasing global population set to reach 9–10 billion in 2050^26^.

As previously reported^20–22^, inhibition of the plant IPCS would lead to a buildup of the enzyme substrate, the Programmed Cell Death (PCD; apoptosis) mediator phytoceramide^27^. The functional divergence of IPCS from the equivalent mammalian enzyme, sphingomyelin synthase (SMS), could allow the identification of specific, non-toxic inhibitors. This possibility has, to date, lead to the identification of 5 potent inhibitors of the fungal IPCS (aureobasidin A^28^ (AbA), khafrefungin^29^, rustimicin^30^, pleofungin^31^ and haplofungin^32^) with low nano-molar IC_50_ values against *Saccharomyces cerevisiae*. However, currently, no inhibitor of the plant orthologue has been identified.

In this study, the well characterized *Arabidopsis thaliana* enzyme *At*IPCS2^20^, the most highly expressed of the 3 IPCS isoforms^33^, was used to complement *S. cerevisiae* lacking *AUR1*. The yeast utilized was engineered to enhance compound sensitivity through reduced expression of several efflux pumps, and thereby allow efficient hit identification in a cell-based high throughput screening (HTS) assay used for 11,440 bioactive compounds. A secondary enzyme-based assay facilitated the validation of hits as inhibitors of the enzyme, and enabled comparison of their activity against the yeast orthologue, Aur1p. This allowed the identification of hits that exhibited selectivity for IPCS2 from *A. thaliana*. The most potent selective compound was tested *in vivo* against seedlings and demonstrated herbicidal activity.

## Results

### Primary high throughput screening using a yeast-based assay

Fungi such as *Saccharomyces cerevisiae* possess multiple genes linked to pleiotropic drug resistance, including those encoding a range of ATP-binding cassette (ABC) transporters and the transcription factors required for their expression^34^. These extrusion pumps can be over-expressed in response to drug treatment, leading to decreased intracellular drug concentrations and subsequent drug resistance^35^, while multiple deletions of these functions render yeast cells significantly more sensitive to a range of toxic compounds including antifungal agents used in agriculture and medicine^8^. To increase the sensitivity of the yeast-based assay platform, an *S. cerevisiae* strain was utilised that lacked *PDR1*, *PDR3*, *PDR16* and *PDR17*. This combination of *pdr* deletions was shown to confer significant hypersensitivity to a range of compounds (Supplementary Information 1). *PDR1*^36^ and *PDR3*^37^ encode paralogous Zn(II)_2_Cys_6_ zinc finger regulators, which control the transcription of ABC drug efflux pump-encoding genes including *PDR5*^38,39^, *SNQ2*^40^, *PDR10*^41^, *PDR15*^41^ and *YOR1*^42^ through binding to *cis*-acting PDREs (pleiotropic drug resistance elements)^40,41,43,44^. *PDR16* and *PDR17* encode a pair of paralogous phosphatidylinositol transport proteins that also confer drug hypersensitivity when deleted^45^.

**Figure 1:**
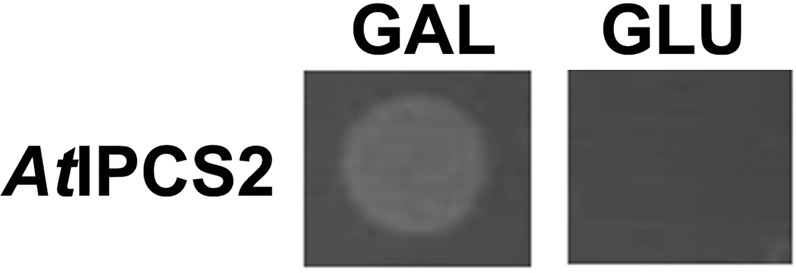
Yeast (MSYD23) dependent on expression of *At*IPCS2 from a galactose inducible promotor were, as expected, viable when grown in the presence of galactose (GAL), but not glucose (GLU). The relevant section of each agar plate (GAL and GLU) is illustrated, the full plates are shown in Supplementary Information 2.

**Figure 2:**
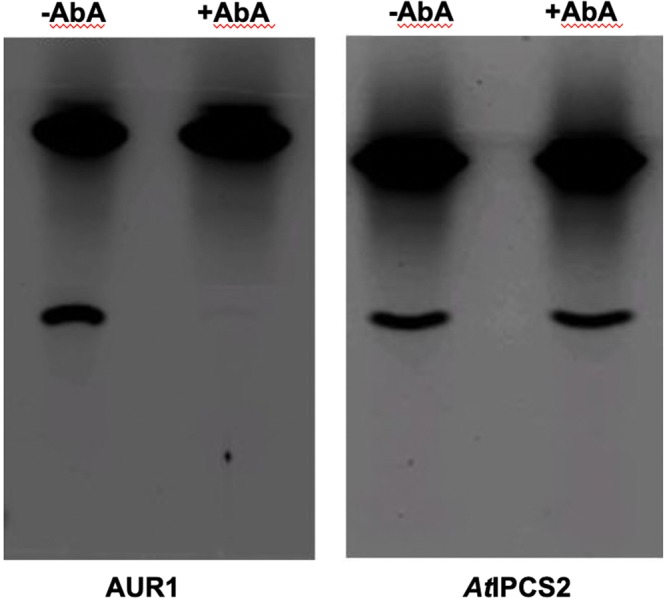
HPTLC separation of *in vitro* assayed Aur1p and *At*IPCS2 showing the production of NBD-IPC in the presence (+) and absence (−) of AbA. Only the fungal enzyme Aur1p is sensitive to the compound. NBD-Cer is the substrate, NBD-C_6_-phytoceramide.

In the quadruple *pdr1∆ pdr3∆ pdr16∆ pdr17∆* strain, *AUR1* was deleted and replaced by a *HIS3* selectable marker, with growth supported by expression of the essential *AUR1* gene from the plasmid pRS316-*AUR1* under uracil selection. In this background, galactose-inducible expression of *At*IPCS2 from plasmid pESC-LEU was found to complement loss of pRS316-*AUR1* when the yeast were cultured in the presence of 5-fluoroorotic acid. This made the yeast dependent upon the presence of galactose for growth, thus demonstrating dependence on the expression of the plant enzyme (Fig. 1, Supplementary Information 2)^20–22^. Assay of microsomal extracts from the complemented yeast demonstrated IPCS activity *in vitro* and confirmed that the plant activity is insensitive to the fungal Aur1p inhibitor aureobasidin A (AbA)^28,33^ (Fig. 2).

**Figure 3:**
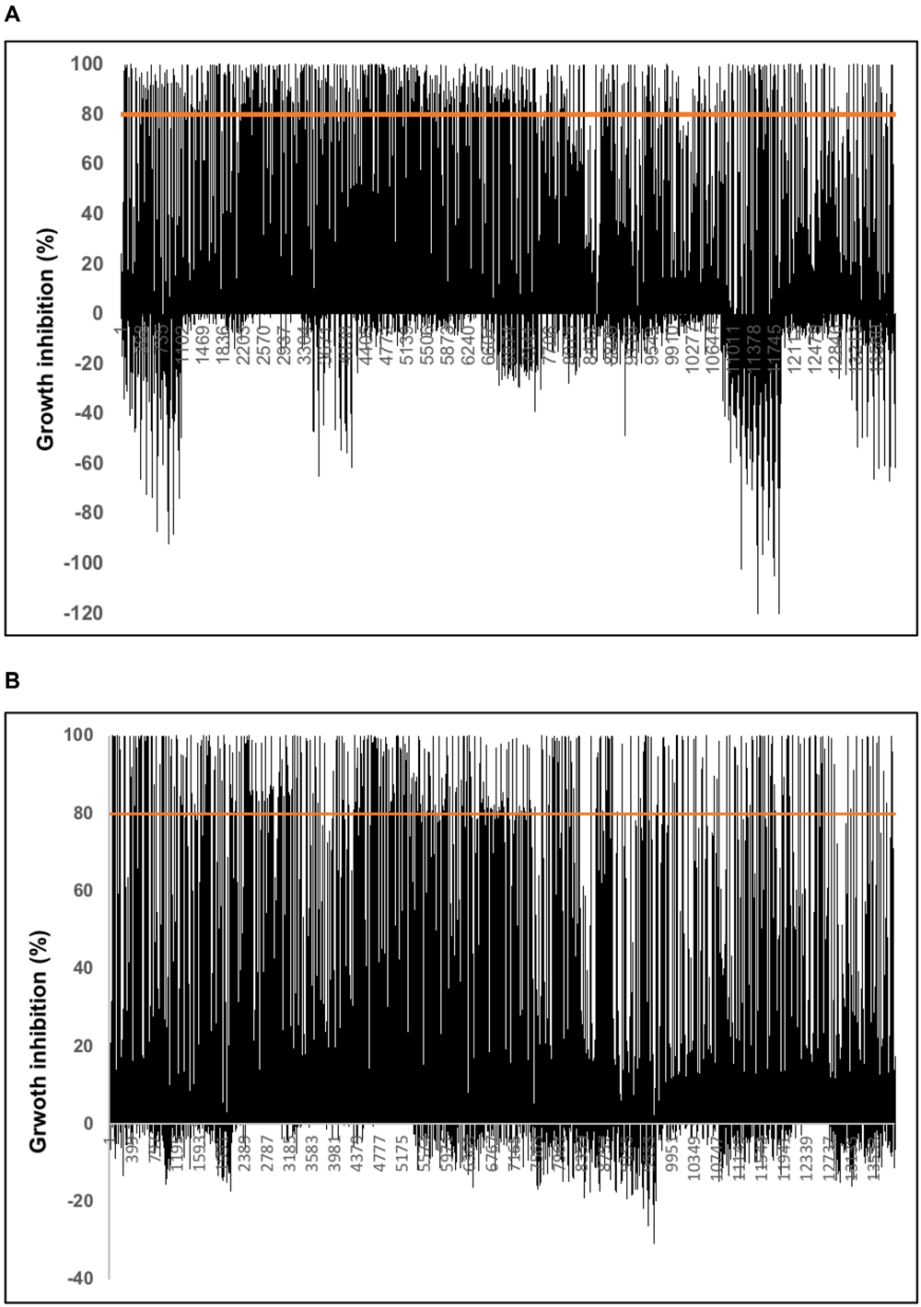
Growth inhibition for compounds (10 µM) against *At*IPCS2 (**A**) and Aur1p (**B**) complemented yeast. After eliminating false positives (2.6%), 106 target directed hits were identified, a hit rate of 0.9%.

Yeast complemented with the well characterised *At*IPCS2, and an *AUR1* control, were subsequently formatted into a 96-well plate. Following statistical validation by calculation of Z factor^46^ in the presence of positive (cycloheximide) and negative (DMSO) controls, the assay was used in HTS of a focused library of 11,440 bioactive compounds. All assay plates were required to have a calculated Z factor ≥0.5 for the data to be progressed. Following in duplicate screening at 10 µM against *At*IPCS2 complemented yeast and the *AUR1* control, compounds exhibiting ≥80% inhibition and ≥50% selectivity for *At*IPCS2 were taken forward. After eliminating false positives (non-reproducible hits; 2.6%), 106 target directed hits were identified, a hit rate of 0.9% (Fig. 3, Supplementary Information 3). It is notable that a significant minority of compounds increased yeast proliferation and that this phenotype was more profound in the *At*IPCS2 complemented yeast (negative inhibition; Fig. 3), whilst this is an interesting observation these were not analysed further. Dose response analyses (50 µM to 68 nM), using the same assay platform, demonstrated that the majority of the inhibitory compound hits (89 of 106) had an IC_50_ of less than 10 µM (Supplementary Information 4).

### Secondary screening using an *in vitro* biochemical assay

**Figure 4:**
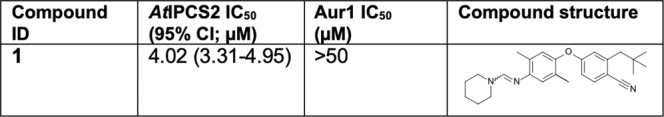
Compound **1** structure and activity in the biochemical assay against *At*IPCS2 and Aur1p.

In the secondary screening stage the previously described microsomal-based *in vitro* IPCS assay was adapted and utilised^17^. Initially, all 106 selective hits from the primary screen were tested, in duplicate, at 10 µM. 16 compounds which, reproducibly, showed ≥30% inhibition were carried forward for in triplicate dose response (100 µM to 46 nM) analyses and IC_50_ determination against *At*IPCS2 and, as a control, *AUR1*. All were active to some degree against the *Arabidopsis* enzyme, whilst none showed inhibition of the fungal orthologue, demonstrating that selective *At*IPCS2 inhibitors had been identified. 4 compounds demonstrated IC_50_ values < 10 µM (Compound **1**, 4.02 µM; **2**, 4.75 µM; **3**, 8.41 µM; and **4**, 9.84 µM; Supplementary Information 5). The structures of compounds **2**–**4** are withheld due to intellectual property reasons, leaving the most active (Compound **1**, a phenylamidine carrying an acetonitrile functional group) to be taken forward (Fig. 4). The structural integrity of Compound **1** was confirmed using mass spectrometry and ^1^H and ^13^C spectroscopy which showed that it was a 3:2 mixture of *E:Z* amidine isomers (see Supplementary Information 6).

### *In vivo* screening

**Figure 5:**
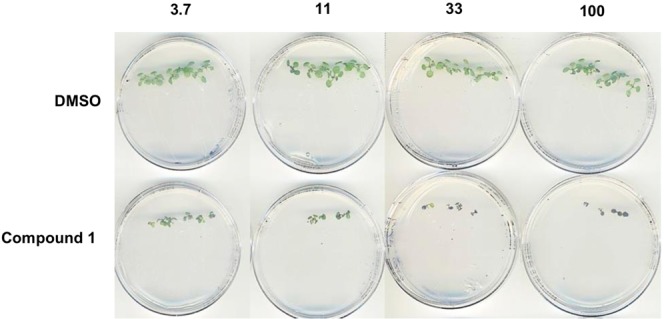
Compound **1** tested against *Arabidopsis* seedlings at 3.7 µM, 11 µM, 33 µM and 100 µM. Treatment restricted growth and led to purple leaf patches at 11 µM and above compared to the DMSO control.

**Figure 6:**
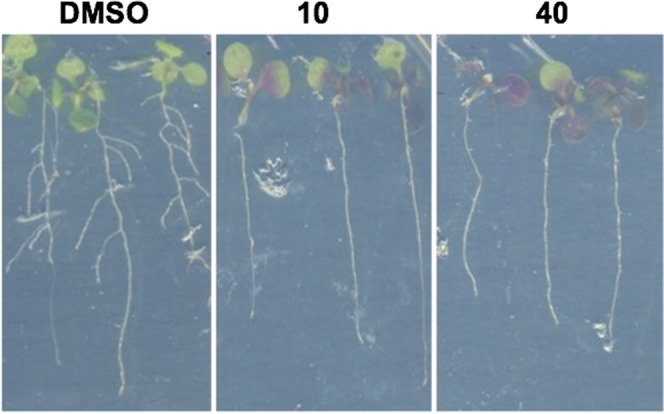
From left to right, 7 day old *Arabidopsis* seedlings grown on agar containing DMSO (vehicle), 10 µM and 40 µM of Compound **1**. Treated plants had clear purple patches and an absence of lateral root development compared to the DMSO control.

*In vivo* testing of the phenylamidine Compound **1** was undertaken against *Arabidopsis* seedlings grown on agar. Dose response analyses (Fig. 5) showed that treatment restricted growth and led to purple leaf patches at 11 µM and above. Examination of treated 7 day old seedlings grown on agar containing 10 and 40 µM of Compound **1** showed plants with clear purple patches associated with anthocyanin biosynthesis in response to stress^47^, and an absence of lateral root development compared to the DMSO control (Fig. 6).

## Discussion

With herbicide resistance increasing^24^ and climate change effecting on crop yield^25^, the need to identify new herbicide targets and lead molecules to address these challenges is pressing. One major hurdle to overcome in this search for a new herbicide is to ensure identified chemicals have acceptable toxicity profiles which are safe to the user and the environment^48^. The divergence in the sphingolipid biosynthetic pathway between mammals and plants, where the former produce SM and the latter IPC^20,33^, may present an opportunity to identify molecules with such a profile.

Following the recent publication of our successful HTS campaign against a protozoan IPCS^49^, this study is the first report of HTS for inhibitors of an enzyme in the plant sphingolipid synthetic pathway, the non-mammalian *At*IPCS2 – the most highly expressed and best characterised isoform in the model dicot *Arabidopsis*, which catalyses the synthesis of IPC^20,33^. The role of this enzyme in phytoceramide homeostasis^20,33^ and therefore PCD^50^, coupled with the product, IPC, functioning as the precursor for the synthesis of glycosylinositol phosphorylceramide (GIPC; 25% of plasma membrane lipid^51^), makes IPCS an attractive target for the discovery of new, non-toxic, herbicidal agents. However, given the multi-transmembrane nature of the enzyme^20,33^ assay development is challenging. Therefore, to facilitate HTS, we developed a novel cell-based assay utilising an *At*IPCS2 complemented *S. cerevisiae* strain lacking 4 extrusion pumps linked to pleiotropic drug resistance (*PDR1*, *PDR3*, *PDR16* and *PDR17*) to increase sensitivity, and utilised this system to screen a library of 11,440 bioactive compounds. Counter screening against Aur1p (the yeast orthologue) formatted in the same assay yielded 106 selective hits, of these 4 demonstrated IC_50_ values < 10 µM in a secondary *in vitro* enzyme assay and minimal activity against yeast Aur1p (>50 µM). The most active was a phenylamidine, Compound **1** (IC_50_ < 5 µM), which has been patented by Bayer as a fungicide^52^. Previous phylogenic analyses^33^ have shown that the three IPCS isoforms in *Arabidopsis* are closely related. Further, focused, sequence analyses demonstrated that whilst *At*IPCS2 orthologues are highly conserved within the monocots and eudicots, there is distance between the two clades (see Supplementary Information 7). This indicated that selective inhibition of the enzyme (for example in a weed species) maybe feasible. Future studies should examine the selectivity of Compound **1** for *At*IPCS2 over the other two isoforms and other plant orthologues to establish selectively in Planta.

The phenylamidines were first identified in the 1960s as pesticides for the control of plant fungal pathogens^52^ and specific variants have subsequently been patented for use as herbicides^53^. *In vivo* screening of the identified phenylamidine, Compound **1**, against wild type Col-0 *Arabidopsis* seedlings demonstrated dose dependent effects with decreased lateral root development, and distinctive purple leaf patches associated with anthocyanin biosynthesis in response to stress^47^. The mode of action of herbicidal phenylamidines have not been published, but the phenotypic effects reported here for Compound **1** are consistent with those expected for an IPCS inhibitor.

In conclusion, using a novel HTS approach the first inhibitor (Compound **1** - a phenylamidine) of plant IPCS was identified and shown, *in vivo*, to induce the plant stress response. This low molecular weight compound is ideal for further development towards use in agriculture, and further studies are planned to investigate this possibility.

## Methods

### Yeast strains

The diploid *Saccharomyces cerevisiae* strain MSYD20 (*MAT***a**/*MATα his3*Δ*1/his3*Δ*1 leu2*Δ*0/leu2*Δ*0 MET15/met15*Δ*0 ura3*Δ*0/ura3*Δ*0 pdr1*∆*::KanMX4/pdr1∆::KanMX4 pdr3∆::KanMX4/pdr3∆::KanMX4 pdr16∆::KanMX4/pdr16∆::KanMX4 pdr17∆::KanMX4/pdr17∆::KanMX4*) is homozygous for knockouts of four genes conferring drug hypersensitivity and was made by sequential crosses and tetrad dissection starting with the single *MAT***a** *pdr1∆::KanMX4* (Y04381), *MATα pdr3∆::KanMX4* (Y13029), *MAT***a** *pdr16∆::KanMX4* (Y01981) and *pdr17∆::KanMX4* (Y11180) strains obtained from the Euroscarf collection (http://www.euroscarf.de). MSYD20 was made heterozygous for *aur1::HIS3* (knocking out the *AUR1* gene encoding yeast IPCS) by transformation with a PCR product amplified from pFA6a-*HIS3* template using the following two primers (plasmid-specific sequences, upper case; *AUR1* flanking sequences, lower case), generating MSYD23.

AUR1-F1

atatcctacaggttgcggttttcatattttaaaaagcttttaatcattcctttgcgtCGGATCCCCGGGTTAATTAA

AUR1-R1

atttatatgtatctacataagaccaaccgtatccgtaattgcagataaaatactcaGAATTCGAGCTCGTTTAAAC

pFA6a-*HIS3* was generated by replacing the *Asc*I-*Mfe*I interval of pFA6a-*TRP1*^54^ that carries the *TRP1* gene with the *Bss*HII-*Eco*RI fragment of YDpH^55^ encoding *HIS3*, enabling amplification of a *HIS3*-containing gene knockout fragment that cannot recombine with existing *KanMX4* gene knockouts such as those present in MSYD20. Sporulation and tetrad analysis of MSYD23 confirmed that *AUR1* is an essential gene since no viable *aur1::HIS3* segregants could be obtained. MSYD23 was next transformed with pRS316-*AUR1*, made by inserting a copy of *AUR1* into the *URA3* plasmid pRS316^56^. Strain MSY23-3C (*MAT***a** *pdr1*Δ*::KanMX, pdr3*Δ*::KanMX4 pdr16*Δ*::KanMX4 pdr17*Δ*::KanMX4 aur1*Δ*::HIS3* [pRS316-*AUR1*]), lacking the four genes conferring drug hypersensitivity and in which growth was supported by *AUR1* expression from pRS316^15^, was identified following sporulation and tetrad analysis of MSYD23 transformed with pRS316-*AUR1*. MSY23-3C was verified by appropriate diagnostic PCR and could not grow in the presence of 5FOA as expected.

### Primary screening, yeast cell-based assay

The complete open reading frames of *AtI*PCS2 and AUR1p were amplified from cDNA or GenEZ ORF Clones (GenScript^®^) using Phusion Flash^®^ PCR master mix (ThermoFisher) according to manufacturer’s guidelines. Primers for In-Fusion® cloning (Clontech) were:

*At*IPCS2F ctcactatagggcccATGACACTTTATATTCGTCGT

*At*IPCS2R tccatgtcgacgcccTCACGCGCCATTCATTGTGTT

AUR1F ctcactatagggcccATGGCAAACC

AUR1R tccatgtcgacgcccTTAAGCCCTC

These open reading frames were cloned into the pESC-LEU vector (Agilent) and verified by sequence analyses, creating pESC-LEU_*At*IPCS2 and pESC-LEU_AUR1. In this vector, expression of the open reading frame was under the control of a galactose-inducible promoter. All plasmids were subsequently transformed into the MSY23-3C *S. cerevisiae* strain as previously described^15^ and selected on SD -TRP -URA -LEU agar (0·17% Bacto yeast nitrogen base, 0·5% ammonium sulphate, 2% glucose, containing the appropriate nutritional supplements) at 30 °C. Yeast were then ‘cured’ of the pRS316-*AUR1* plasmid by selection on SGR -TRP –LEU +FOA agar (0·17% Bacto yeast nitrogen base, 0·5% ammonium sulphate, 0.1% galactose, 1% raffinose, 0.1% 5-Fluoroorotic Acid Monohydrate (FOA) containing the appropriate nutritional supplements) at 30 °C, creating MSY23-3C pESC-LEU_*At*IPCS2; and MSY23-3C pESC-LEU_AUR1. Following PCR validation and propagation in SGR -TRP -LEU, frozen stocks of both yeast lines were created (OD_600_ = 10).

When required, MSY23-3C pESC-LEU_*At*IPCS2 and MSY23-3C pESC-LEU_AUR1 were thawed on ice and diluted 1:20 with SGR -TRP -LEU. Using a Biomek FX^p^ automated workstation (Beckman Coulter) 198 µl was aliquoted into 96-well plates (Thermo Scientific) before the addition of 2 ul of compounds (to the desired concentration) and controls – DMSO (negative; Sigma Aldrich) and cycloheximide (to 10 µM; positive; Sigma Aldrich). Following incubation at 30 °C for 24 hours optical density (OD_600_) was measured (Biotek Synergy H4 with Gen5™). All assays were carried out in duplicate and inhibition (%) calculated.

### Secondary screening, biochemical assay

Microsomal material was prepared from MSY23-3C pESC-LEU_*At*IPCS2 and MSY23-3C pESC-LEU_AUR1 as previously described^33^. IPCS turnover was assayed using HPTLC (Merck) and imaged using a Fuji FLA−3000 reader and AIDA Image Analyser® software (version 3.52) as previously described^17^. Subsequently, a 96-well plate assay was formatted based on the protocol described by Mina *et al*.^17^. Following optimisation of substrate concentration and incubation time, each compound at the desired concentration (100 µM to 46 nM; in triplicate), was incubated in 96-well plates (Corning^®^ Costar^®^) in phosphate buffer (71.4 mM, pH 7.0) with soybean PI (100 µM, final concentration, Avanti), NBD-C_6_-phytoceramide (15 µM; ThermoFisher) and microsomal membranes (0.3–0.4 Units^17^). Following incubation for 60 minutes (or 40 minutes for MSY23-3C pESC-LEU_AUR1 membranes) at 30 °C the reaction was quenched by the addition of 200 µl methanol per well, the reaction product separated using exchange chromatography in 96-well filter plates (Millipore)^17^ and the fluorescence measured at Ex460/Em540 using a fluorescence Microplate Reader (Biotek Synergy H4 with Gen5™). Analyses were carried out using GraphPad Prism 7.

### *In vivo* screening, Arabidopsis seedlings

*A. thaliana* (Col0) seedlings were grown for 10 days on 0.8% Murashige and Skoog (MS) agar and then transferred to 1.2% MS agar containing compounds at the desired concentrations or DMSO as a control. Plants were grown at 20 °C under 16 hour day/8 hour night photoperiod.

## Supplementary information

Supplementary Information
