# Supplementary material for: The identification of small molecule inhibitors of the plant inositol phosphorylceramide synthase which demonstrate herbicidal activity
Source: Sci Rep. 2019 May 30;9:8083. doi: 10.1038/s41598-019-44544-1 (PMC6542793; doi:10.1038/s41598-019-44544-1)
Supplement: Supplementary file 1 — Supplementary Information [file 41598_2019_44544_MOESM1_ESM.pdf]

The identification of small molecule inhibitors of the plant inositol phosphorylceramide synthase which demonstrate herbicidal activity

Elizabeth C. Pinneh,[1] John G. Mina[2], Michael J.R. Stark,[3] Stephen D. Lindell[4], Peter Luemmen[4], Marc R. Knight[2], Patrick G. Steel[1*] and Paul W. Denny[2*]

Supplementary Information 1

The sensitivity of different yeast strains to 320 compounds from the NCI Diversity set provided by the Drug Synthesis and Chemistry Branch, Developmental Therapeutics Program (Division of Cancer Treatment and Diagnosis, NIH) was examined by looking for growth inhibition of yeast cells cultured in 96-well plate format using plates with flat-bottomed wells. Each well was inoculated with 100 µl YPD medium (per litre 10 g yeast extract, 20 g peptone, 20 g glucose) containing the indicated yeast strain at a concentration of 10^5 cells/ml. Wells in row 1 (top row of each plate in the Figure) received 1 µl DMSO as a control, while wells in rows 2-11 each received 1 µl of a 1 mM solution in DMSO of one of the 80 compounds from a plate of compounds chosen arbitrarily from the NIH Diversity Set, giving a final concentration of 10 µM. The assay plates were incubated at 26°C for 24 hours and then each plate photographed after gentle mixing to resuspend the yeast cells. The Figure shows a montage of 96-well plate photographs assembled from three separate experiments as demarcated by the grey lines (note in each case that row 12 of the plate is not shown). Plates in each column contained the same indicated strain, while each row of plates received compounds from the same plate of compounds (set 1, compound plate 1410; set 2, compound plate 1417; set 3, compound plate 1401; set 4, compound plate 1402).

Wells showing substantial growth inhibition as indicated by reduced or absent cell density after incubation are highlighted by red circles in the Figure. Only 3/320 compounds inhibited growth of the control strain, while 32/320 inhibited growth of the quadruple *pdr1Δ pdr3Δ pdr16Δ pdr17Δ* strain indicating elevated drug hypersensitivity. The two double mutant combinations (*pdr1Δ pdr3Δ* and *pdr16Δ pdr17Δ*) each showed sensitivity to more compounds than the wild-type strain but to less than the quadruple mutant used in the *At*IPCS2 screen.

The yeast strains used were as follows: Wild-type BY4742: *MATα his3Δ1 leu2Δ0 lys2Δ0 ura3Δ0*); *pdr1Δ pdr3Δ* (MSY510-8A: *MATα his3Δ1 leu2Δ0 met15Δ0 ura3Δ0 pdr1Δ::KanMX4 pdr3Δ::KanMX4*); *pdr16Δ pdr17Δ* (MSY512-16D: *MAT***a** *his3Δ1 leu2Δ0 met15Δ0 ura3Δ0 pdr16Δ::KanMX4 pdr17Δ::KanMX4*), *pdr1Δ pdr3Δ pdr16Δ pdr17Δ* (CJY13-10A: *MAT***a** *his3Δ1 leu2Δ0 lys2Δ0 ura3Δ0 pdr1Δ::KanMX4 pdr3Δ::KanMX4 pdr16Δ::NatRMX pdr17Δ::NatRMX*).

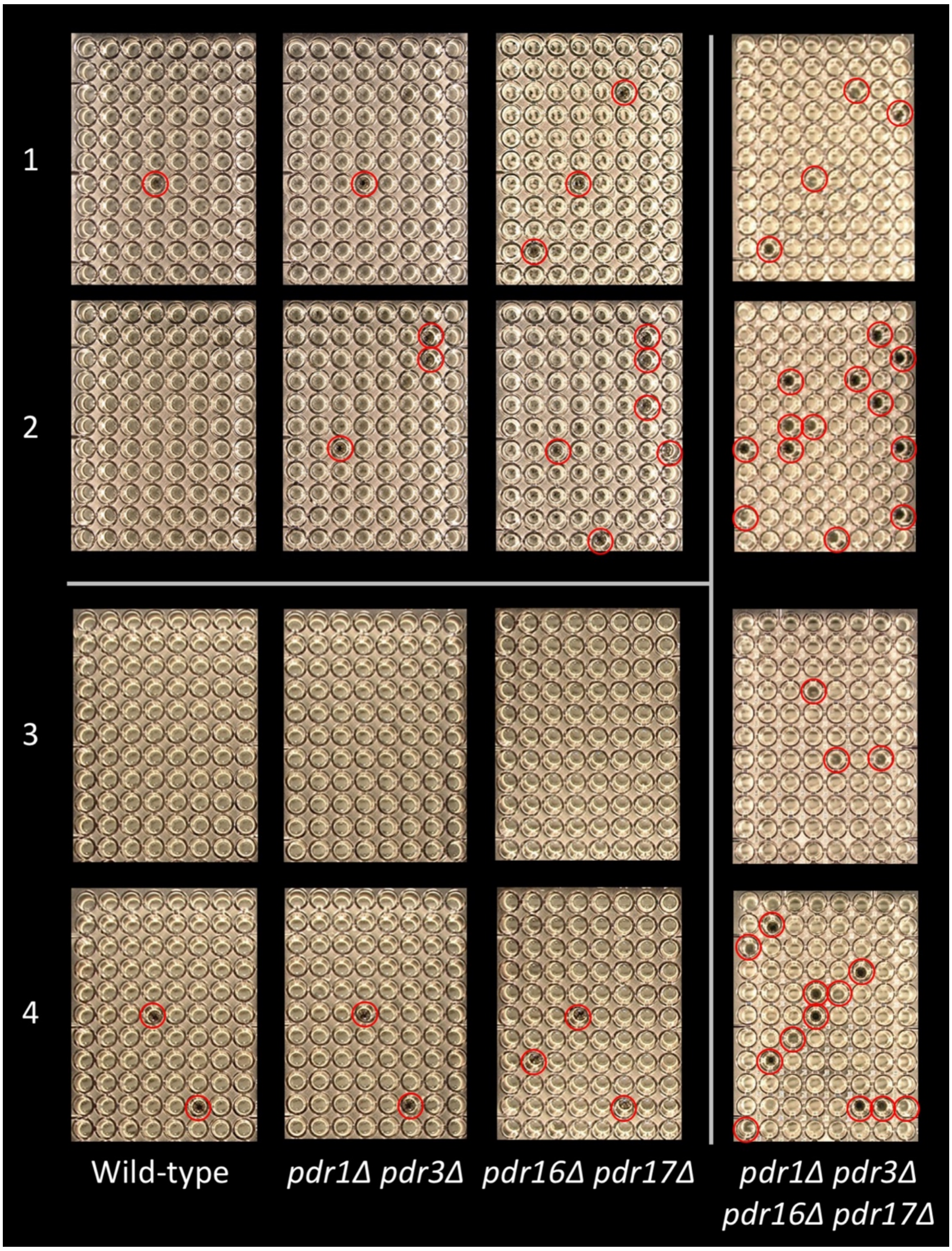
1
2
3
4
Wild-type
pdr1Δ pdr3Δ
pdr16Δ pdr17Δ
pdr1Δ pdr3Δ
pdr16Δ pdr17Δ

Supplementary Information 2

Yeast (MSYD23) dependent on expression of *At*IPCS1, *At*IPCS2 and *At*IPCS3 from a galactose inducible promotor were, as expected, viable when grown in the presence of galactose (GAL), but not glucose (GLU). Subsequent focus was on *At*IPCS2, the most highly expressed isoform in *Arabidopsis.*

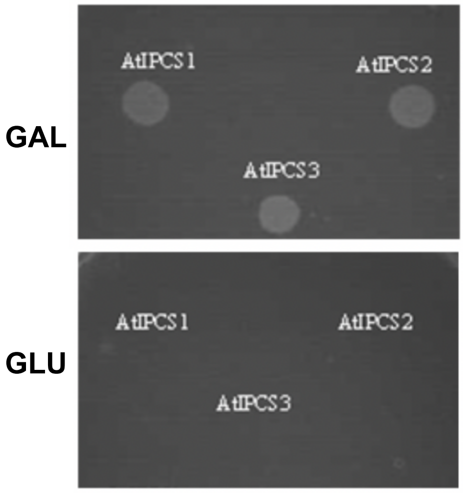

Supplementary Information 3

Plot of the 106 hit compounds that demonstrated $\geq$80% inhibition and $\geq$50% selectivity for *At*IPCS2 over Aur1p in the primary screen. Compounds were screened at 10 µM. Data is mean of n=2.

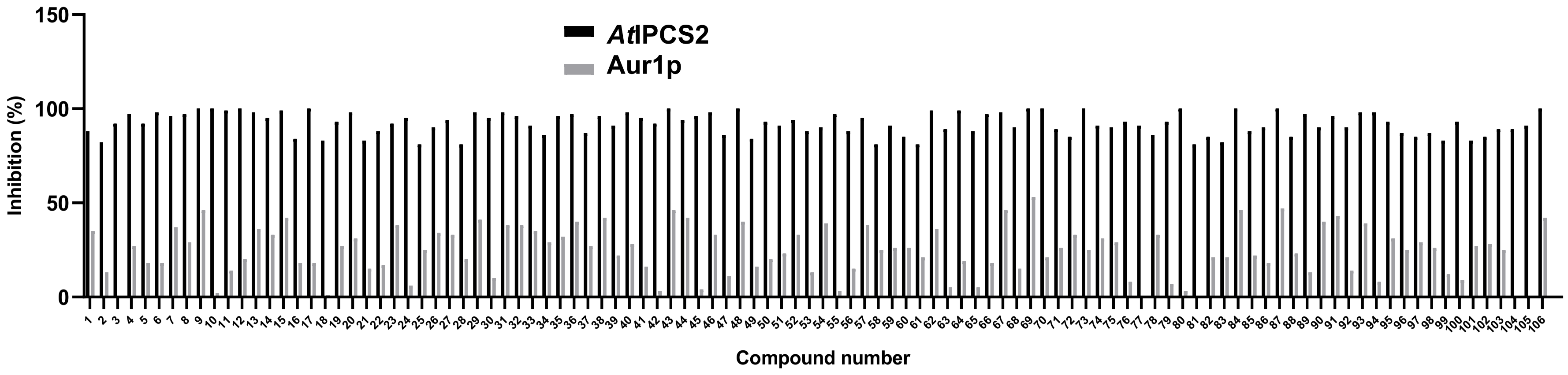
150
100
50
0
Inhibition (%)
AtIPCS2
Aur1p
1 2 3 4 5 6 7 8 9 10 11 12 13 14 15 16 17 18 19 20 21 22 23 24 25 26 27 28 29 30 31 32 33 34 35 36 37 38 39 40 41 42 43 44 45 46 47 48 49 50 51 52 53 54 55 56 57 58 59 60 61 62 63 64 65 66 67 68 69 70 71 72 73 74 75 76 77 78 79 80 81 82 83 84 85 86 87 88 89 90 91 92 93 94 95 96 97 98 99 100 101 102 103 104 105 106
Compound number

Supplementary Information 4

IC_{50} curves of the 106 compounds (A-K) which showed selective inhibition of *At*IPCS2 complemented yeast over Aur1p complemented yeast. Compounds were tested at a starting concentration of 50 μM and serially diluted (1:3) to a final concentration of 68 nM. Compound inhibition was determined by measuring optical density of cells at 600 nm following 24 hours incubation with compound at 30°C, and relative inhibition calculated compared with controls. 89 of the 106 compounds had an IC_{50} of less than 10 μM. Data is mean of n=2 with standard deviation indicated. Graphs were plotted using GraphPad Prism 7 and [Inhibitor] vs. response (variable slope four parameter equation).

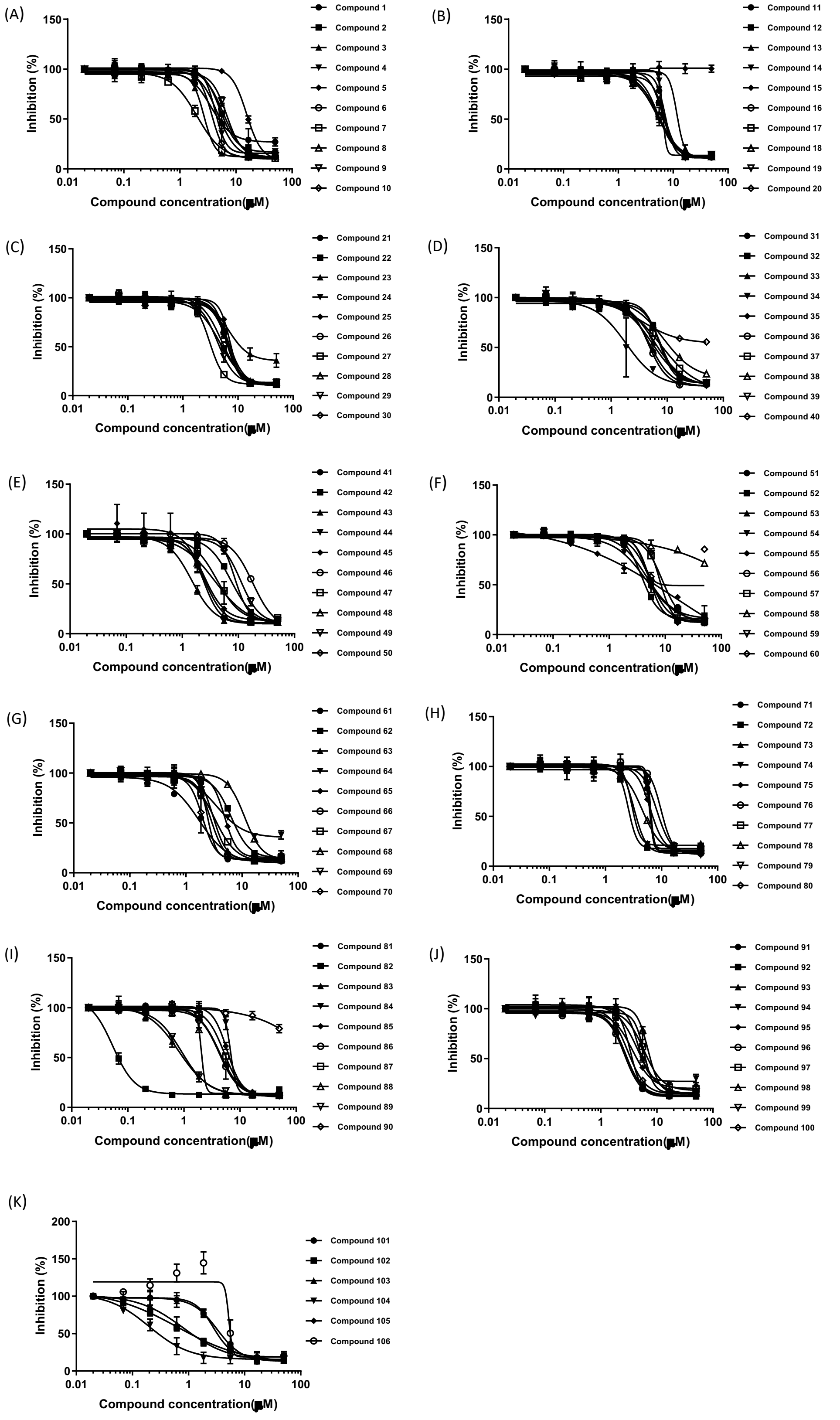
(A)
Inhibition (%)
Compound concentration(μM)
Compound 1
Compound 2
Compound 3
Compound 4
Compound 5
Compound 6
Compound 7
Compound 8
Compound 9
Compound 10
(B)
Compound 11
Compound 12
Compound 13
Compound 14
Compound 15
Compound 16
Compound 17
Compound 18
Compound 19
Compound 20
(C)
Compound 21
Compound 22
Compound 23
Compound 24
Compound 25
Compound 26
Compound 27
Compound 28
Compound 29
Compound 30
(D)
Compound 31
Compound 32
Compound 33
Compound 34
Compound 35
Compound 36
Compound 37
Compound 38
Compound 39
Compound 40
(E)
Compound 41
Compound 42
Compound 43
Compound 44
Compound 45
Compound 46
Compound 47
Compound 48
Compound 49
Compound 50
(F)
Compound 51
Compound 52
Compound 53
Compound 54
Compound 55
Compound 56
Compound 57
Compound 58
Compound 59
Compound 60
(G)
Compound 61
Compound 62
Compound 63
Compound 64
Compound 65
Compound 66
Compound 67
Compound 68
Compound 69
Compound 70
(H)
Compound 71
Compound 72
Compound 73
Compound 74
Compound 75
Compound 76
Compound 77
Compound 78
Compound 79
Compound 80
(I)
Compound 81
Compound 82
Compound 83
Compound 84
Compound 85
Compound 86
Compound 87
Compound 88
Compound 89
Compound 90
(J)
Compound 91
Compound 92
Compound 93
Compound 94
Compound 95
Compound 96
Compound 97
Compound 98
Compound 99
Compound 100
(K)
Compound 101
Compound 102
Compound 103
Compound 104
Compound 105
Compound 106

Supplementary Information 5

IC_{50} curves of the 4 compounds (the numbers equate to those in Supplementary Information 4) which showed significant inhibition (IC_{50} <10 µM) of *At*IPCS2 in the secondary, 96-well plate based, enzyme assay. None showed activity against Aur1p in the same platform. Compounds were tested at a starting concentration of 100 µM and serially diluted (1:3) to a final concentration of 46 nM. Data is mean of n=3 with standard deviation indicated. Graphs were plotted using GraphPad Prism 7 and [Inhibitor] vs. response (variable slope four parameter equation).

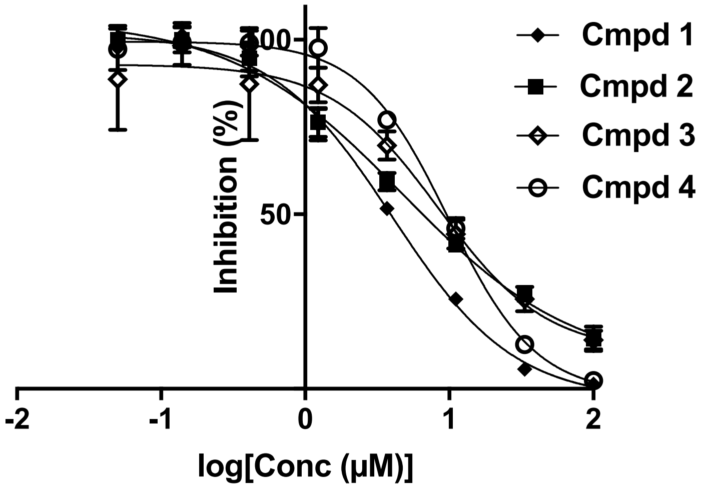

Supplementary Information 6

A.

Analytical HPLC was conducted under neutral conditions using a Waters 2695 apparatus equipped with a reverse phase Triart C18 column (150 x 4.6 mm); gradient elution with MeCN/H_2O; UV detector (210 nm). The purity of Compound 1 was ascertained to be 91%, inset table shows relative quantity of each peak. RT – Retention Time

B.

Accurate mass of major peak from A measured using a Waters Q-ToF Premier apparatus under electrospray ionization conditions. m/z $[MH]^+$ measured for major peak was 404.2695, within 1.7 parts per million of the calculated value (404.2702) for Compound **1 -** $C_{26}H_{34}N_3O$.

C.

NMR spectra were measured in DMSO-d_6 using a Brucker Advance III 600 MHz spectrometer with TMS as an internal standard. The ^{1}H NMR spectrum indicated that Compound 1 is a mixture of *E:Z* amidine isomers in a 3:2 ratio, respectively, as indicated by the doubling-up of many resonance signals: δ 0.89, 0.90 (2s, 9H, t-Bu), 1.40-1.62 (m, 6H, 3 x piperidyl-CH_2), 2.00, 2.06, 2.12, 2.18 (4s, 6H, 2 x aryl-CH_3), 2.65, 2.67 (2S, 2H, CH_2), 3.30-3.60 (bm, 4H, 2 x piperidyl-CH_2), 6.75-6.84 (m, 2H, 2 x aryl-H), 7.45 (s, 0.4H, amidine-CH, *Z*-isomer), 7.48 (m, 1H, aryl-H), 7.61 (s, 0.6H, amidine-CH, *E*-isomer). 7.74, 7.80 (2m, 2H, 2 x aryl-H).

A

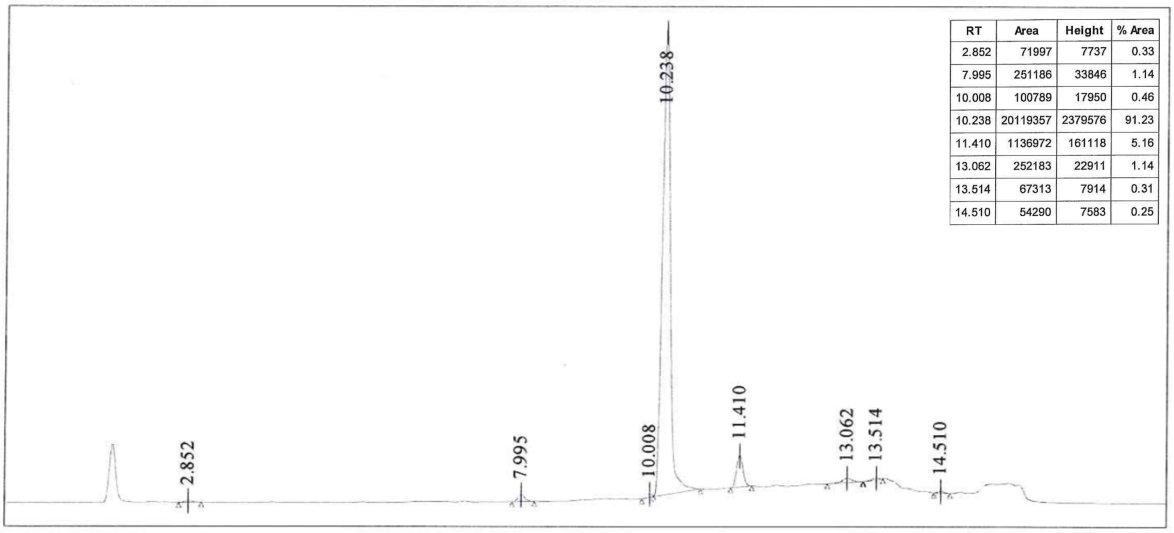

RT	Area	Height	% Area
2.852	71997	7737	0.33
7.995	251186	33846	1.14
10.008	100789	17950	0.46
10.238	20119357	2379576	91.23
11.410	1136972	161118	5.16
13.062	252183	22911	1.14
13.514	67313	7914	0.31
14.510	54290	7583	0.25

B

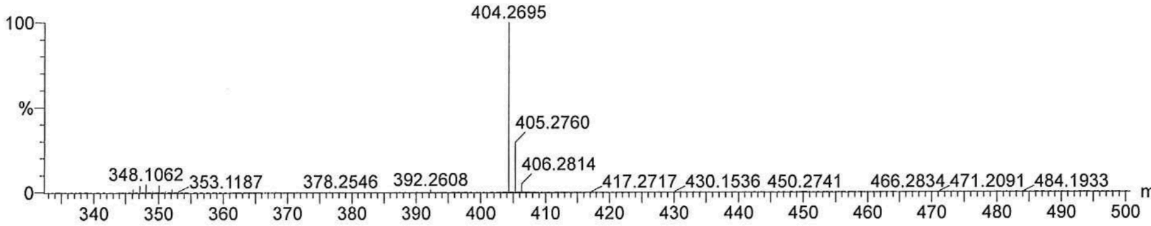

C

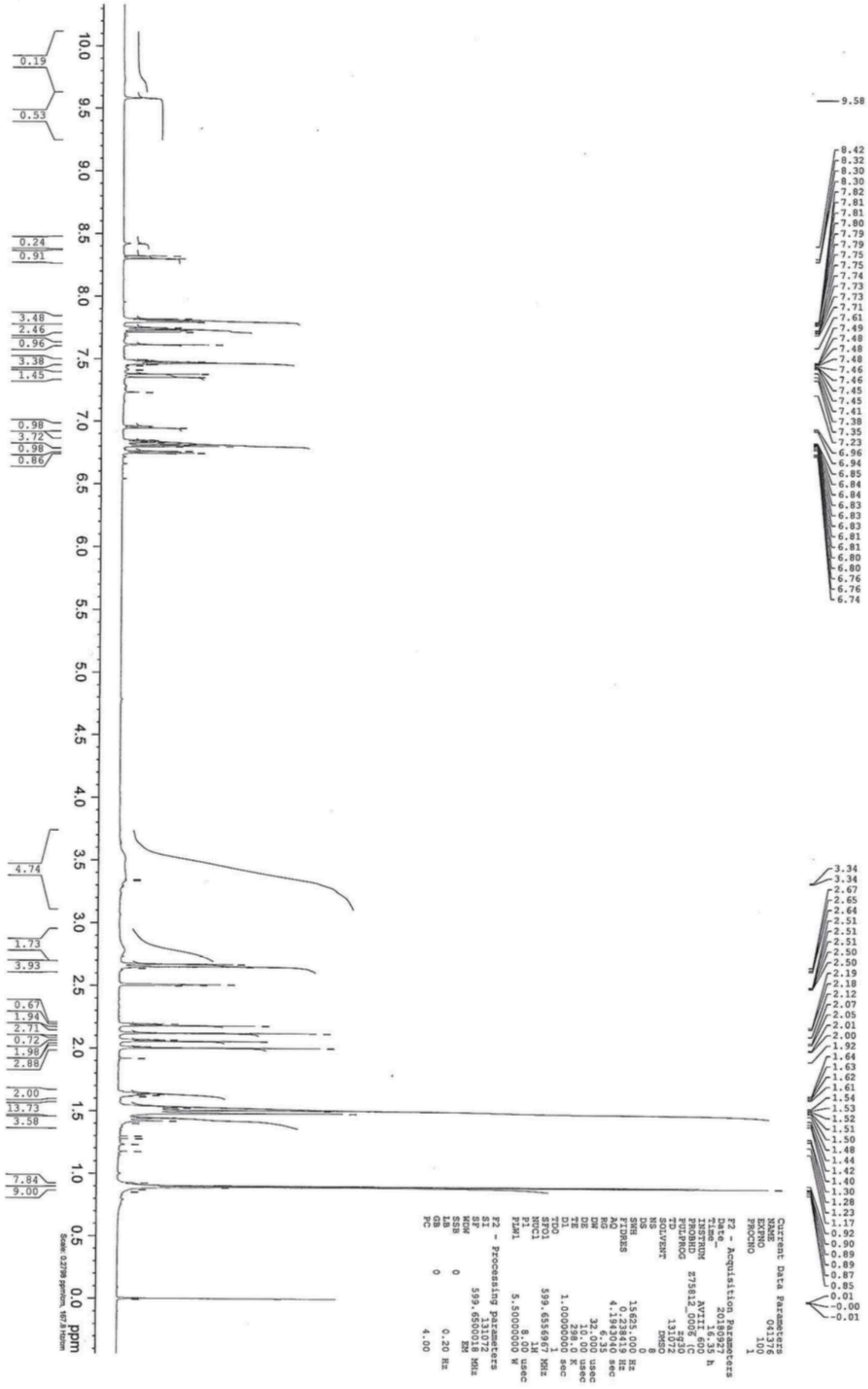
ppm
Current Data Parameters
NAME 041376
EXPNO 100
PROCNO 1
F2 - Acquisition Parameters
Date_ 20180927
Time 16.35 h
INSTRUM AVIII 600
PROBHD Z75812_0006 (C
PULPROG zg30
TD 131072
SOLVENT DMSO
NS 8
DS 0
SWH 15625.000 Hz
FIDRES 0.238419 Hz
AQ 4.1943040 sec
RG 6.35
DW 32.000 usec
DE 10.00 usec
TE 298.0 K
D1 1.00000000 sec
TD0 1
SFO1 599.6556967 MHz
NUC1 1H
P1 8.00 usec
PLW1 5.50000000 W
F2 - Processing parameters
SI 131072
SF 599.6500018 MHz
WDW EM
SSB 0
LB 0.20 Hz
GB 0
PC 4.00

Supplementary Information 7

Comparison of the protein sequence of *At*IPCS2 with orthologues from both eudicot and monocot plants, using Clustal Omega alignment (www.ebi.ac.uk). A. Tree produced using the Simple Phylogeny tool (Distance Matrix using UPGMA clustering, distance correction and excluding gaps). Species names and associated accession numbers are indicated. B. Percent Identity Matrix from the same alignment data.

A

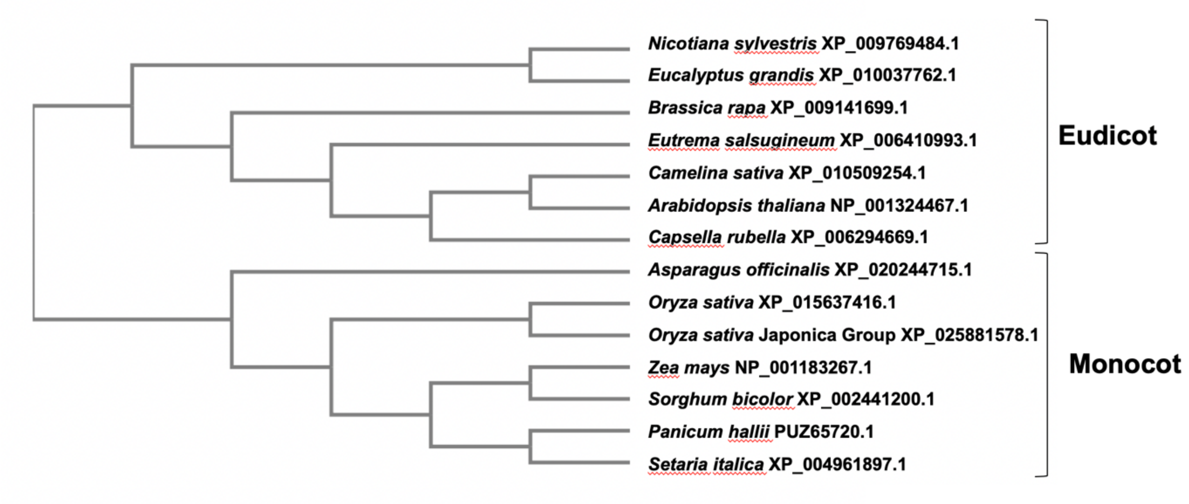

B

		1	2	3	4	5	6	7	8	9	10	11	12	13	14
Eudicot	1: *Nicotiana sylvestris*	100.00	82.95	78.07	77.70	78.74	78.41	78.07	68.71	67.43	68.33	69.54	68.87	69.64	69.54
	2: *Eucalyptus grandis*	82.95	100.00	80.66	82.33	82.30	81.64	81.97	72.58	69.87	70.59	70.00	69.35	70.42	70.32
	3: *Brassica rapa*	78.07	80.66	100.00	95.67	93.44	94.75	93.77	69.87	67.43	68.68	68.21	67.88	67.33	67.55
	4: *Eutrema salsugineum*	77.70	82.33	95.67	100.00	97.00	97.00	97.33	69.80	67.56	68.84	70.37	70.03	69.13	69.70
	5: *Camelina sativa*	78.74	82.30	93.44	97.00	100.00	97.38	97.70	70.53	68.42	69.75	69.87	69.54	69.31	69.87
	6: *Arabidopsis thaliana*	78.41	81.64	94.75	97.00	97.38	100.00	97.70	70.53	68.09	69.40	70.53	70.20	69.31	69.87
	7: *Capsella rubella*	78.07	81.97	93.77	97.33	97.70	97.70	100.00	69.87	67.43	68.68	69.54	69.21	68.32	68.87
Monocot	8: *Asparagus officinalis*	68.71	72.58	69.87	69.80	70.53	70.53	69.87	100.00	79.87	82.11	79.74	79.41	79.80	80.72
	9: *Oryza sativa*	67.43	69.87	67.43	67.56	68.42	68.09	67.43	79.87	100.00	100.00	89.34	89.03	91.25	91.85
	10: *Oryza sativa Japonica*	68.33	70.59	68.68	68.84	69.75	69.40	68.68	82.11	100.00	100.00	89.86	89.19	91.58	92.23
	11: *Zea mays*	69.54	70.00	68.21	70.37	69.87	70.53	69.54	79.74	89.34	89.86	100.00	96.55	94.36	95.92
	12: *Sorghum bicolor*	68.87	69.35	67.88	70.03	69.54	70.20	69.21	79.41	89.03	89.19	96.55	100.00	94.67	96.24
	13: *Panicum hallii*	69.64	70.42	67.33	69.13	69.31	69.31	68.32	79.80	91.25	91.58	94.36	94.67	100.00	97.81
	14: *Setaria italica*	69.54	70.32	67.55	69.70	69.87	69.87	68.87	80.72	91.85	92.23	95.92	96.24	97.81	100.00
